# Supplementary material for: Origin and evolution of the nuclear auxin response system
Source: eLife. 2018 Mar 27;7:e33399. doi: 10.7554/eLife.33399 (PMC5873896; doi:10.7554/eLife.33399)
Supplement: Supplementary file 2. [file elife-33399-supp2.pptx]

## Slide 1
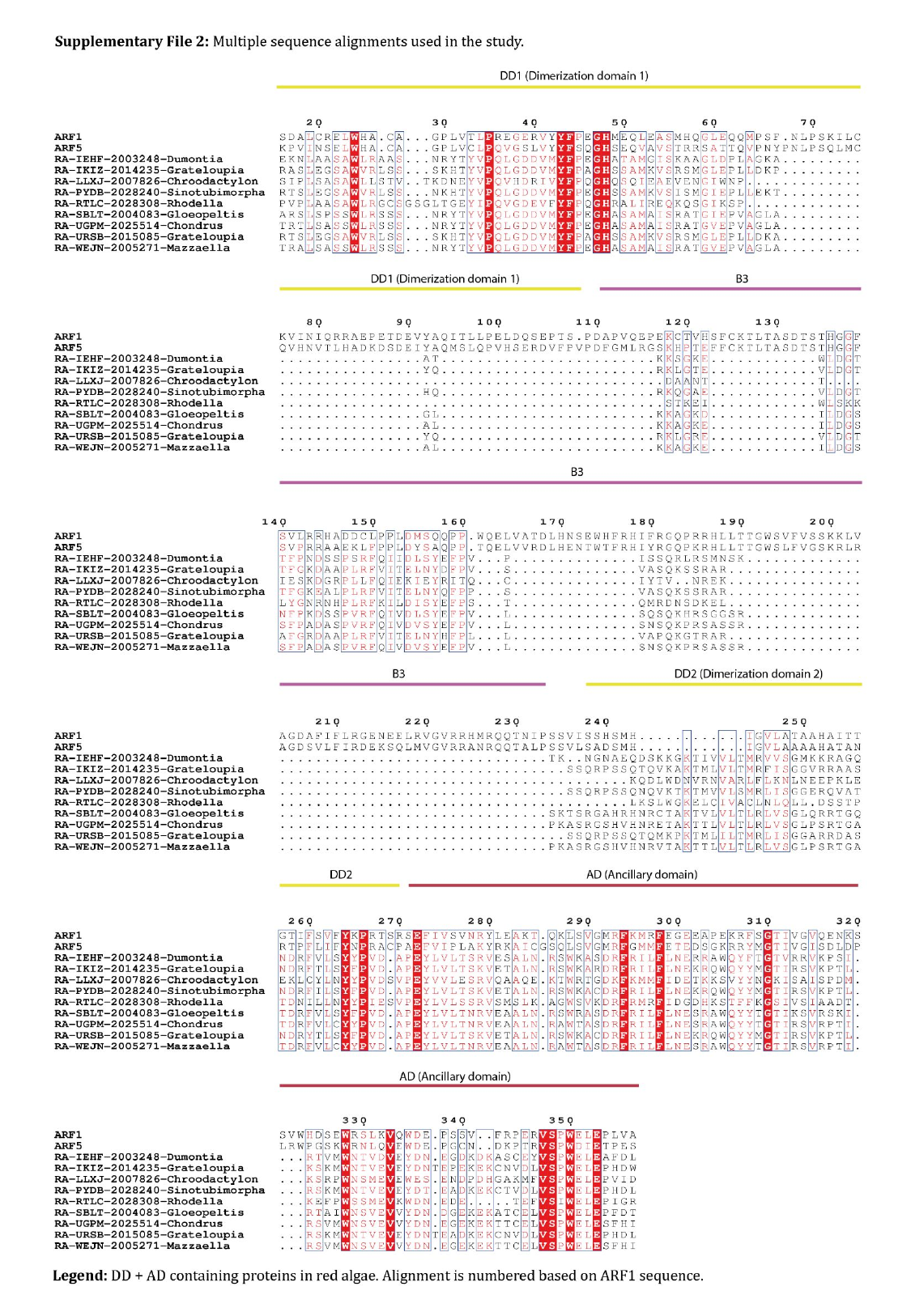

## Slide 2
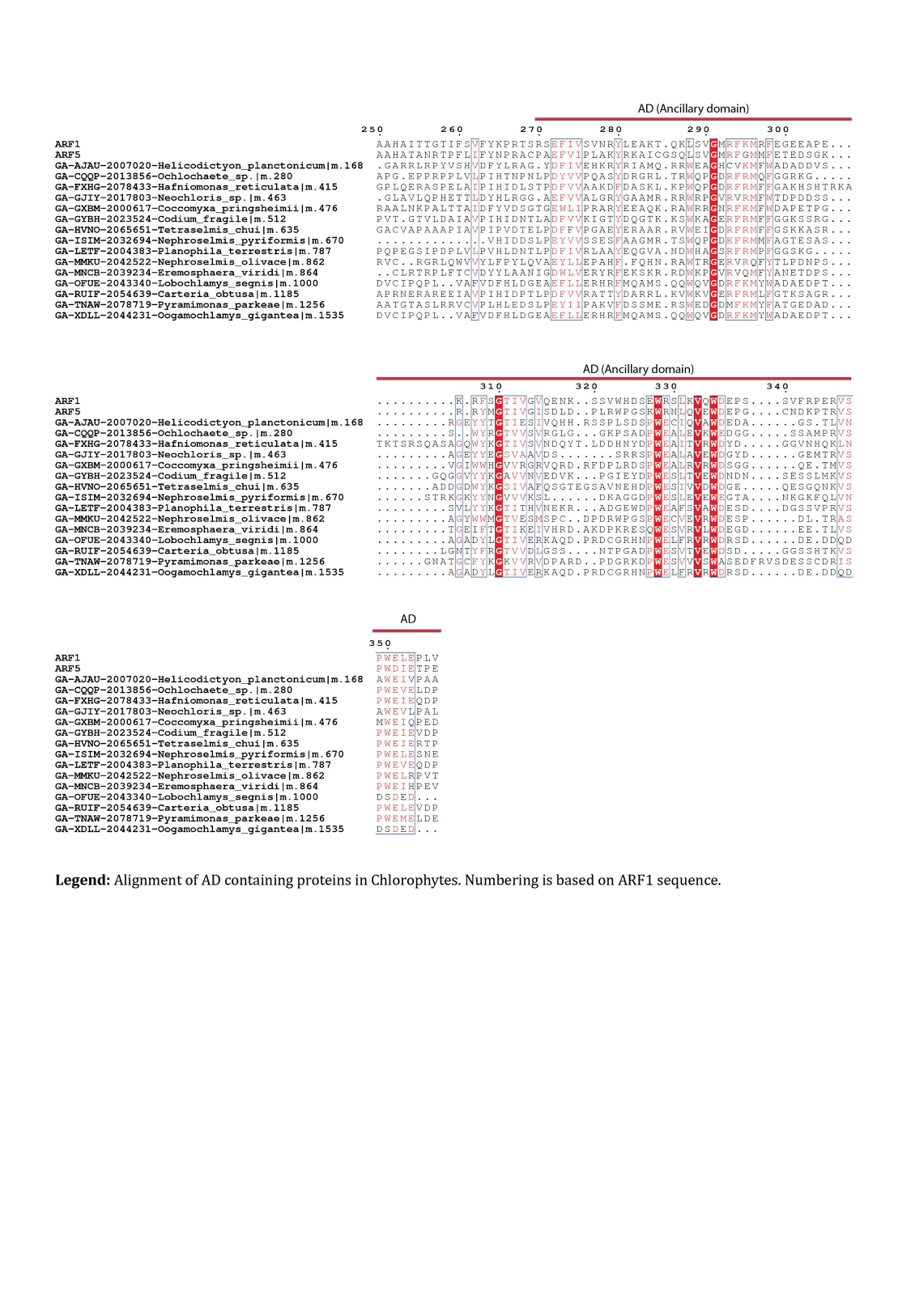

## Slide 3
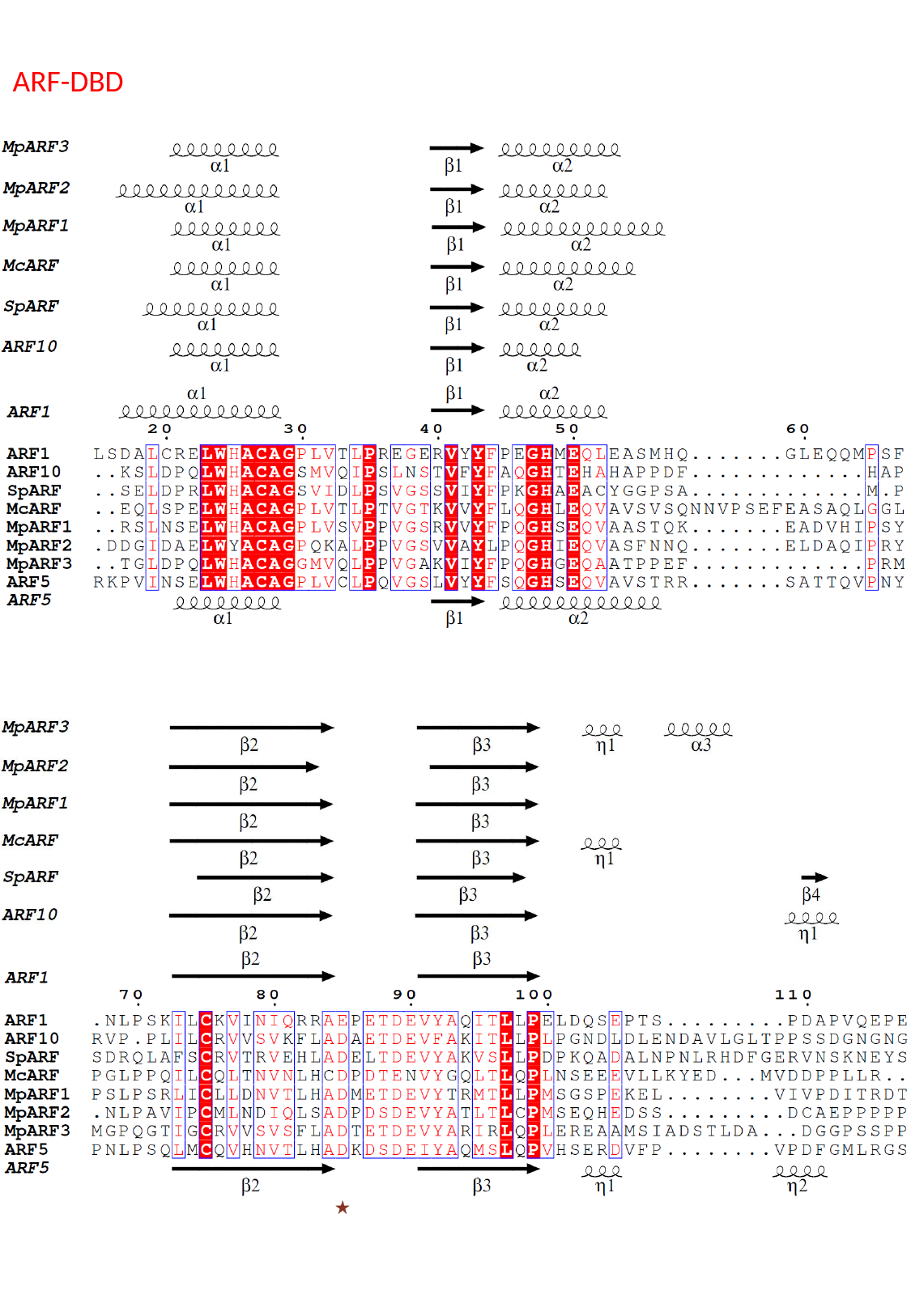

ARF-DBD

## Slide 4
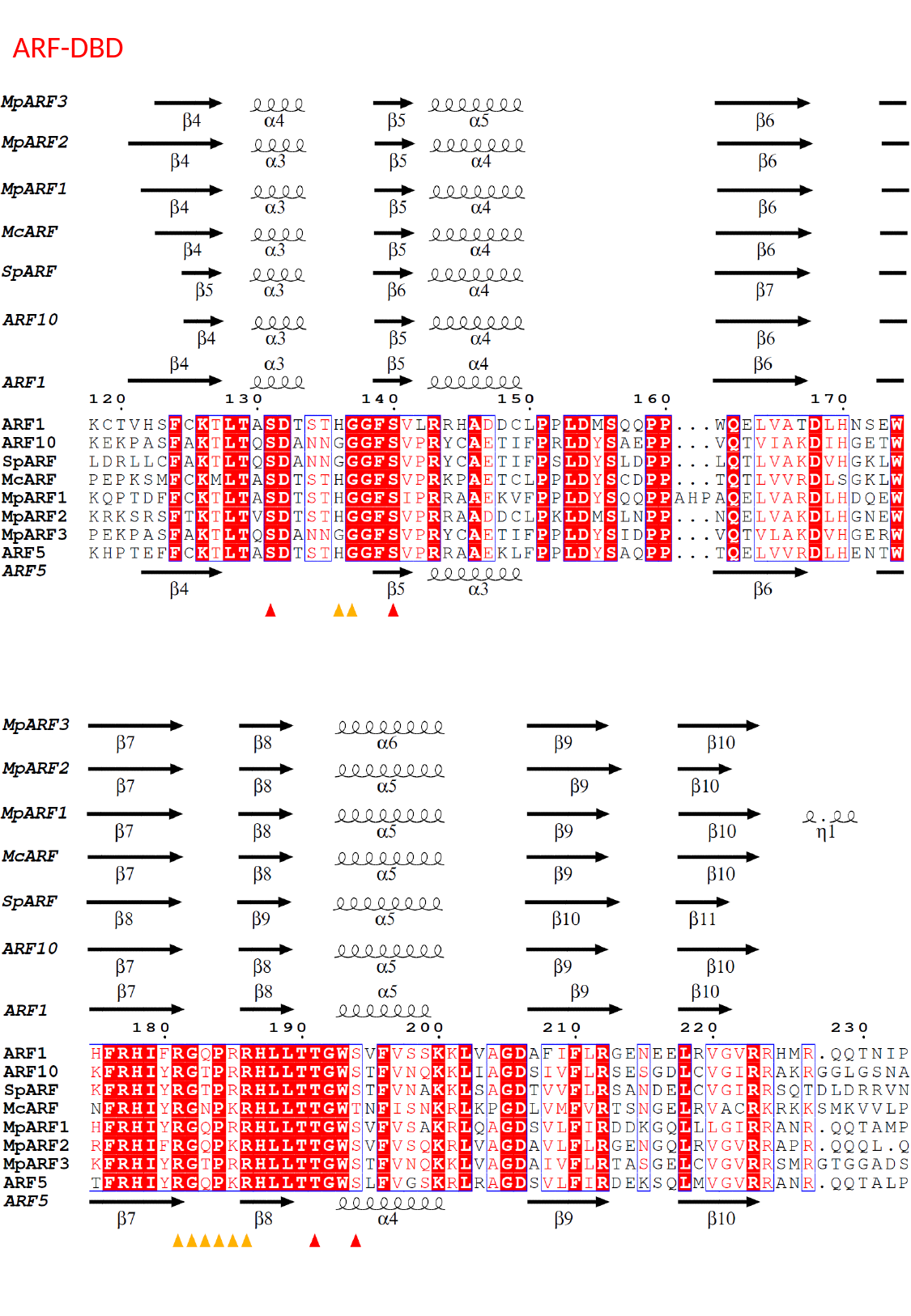

ARF-DBD

## Slide 5
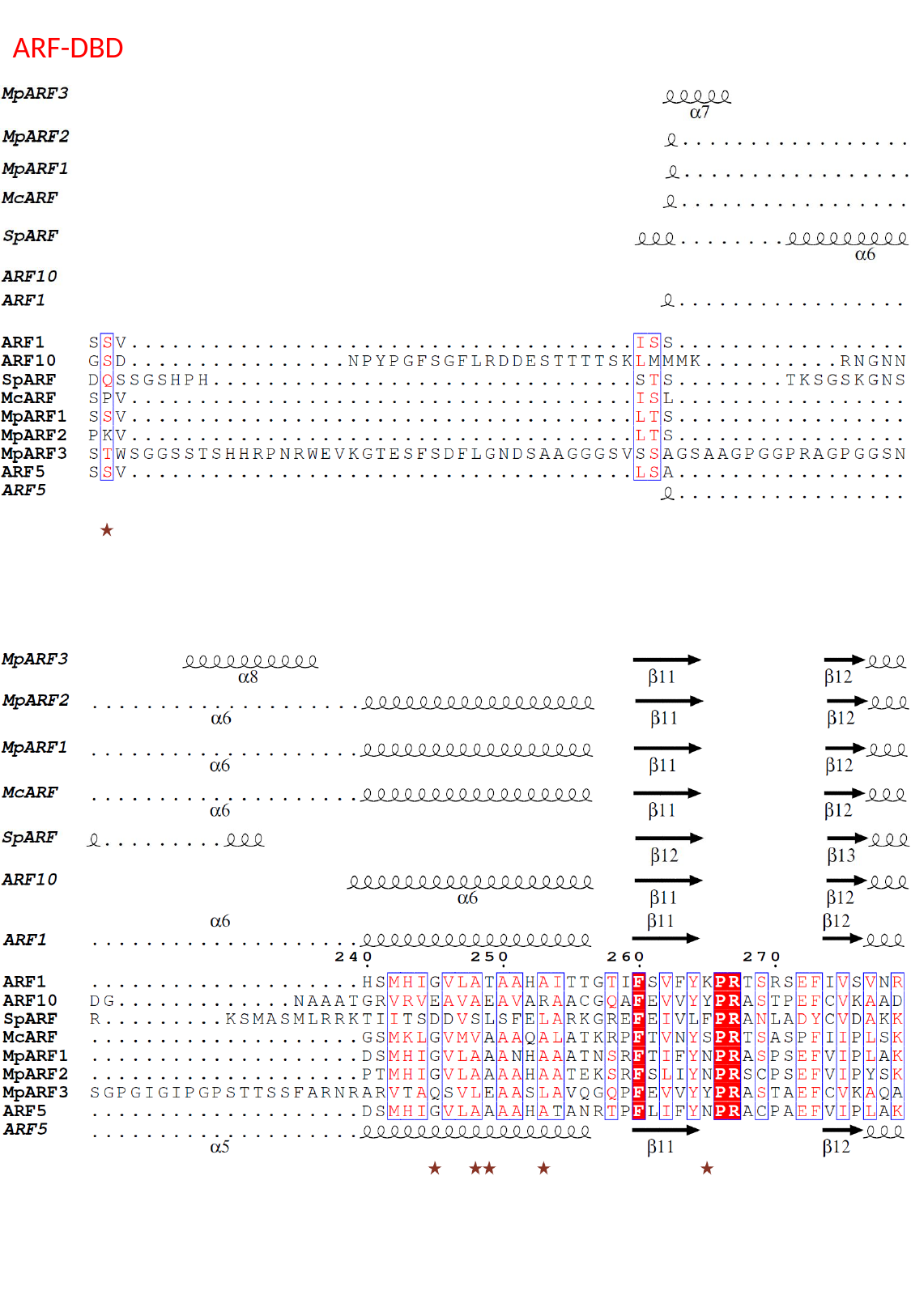

ARF-DBD

## Slide 6
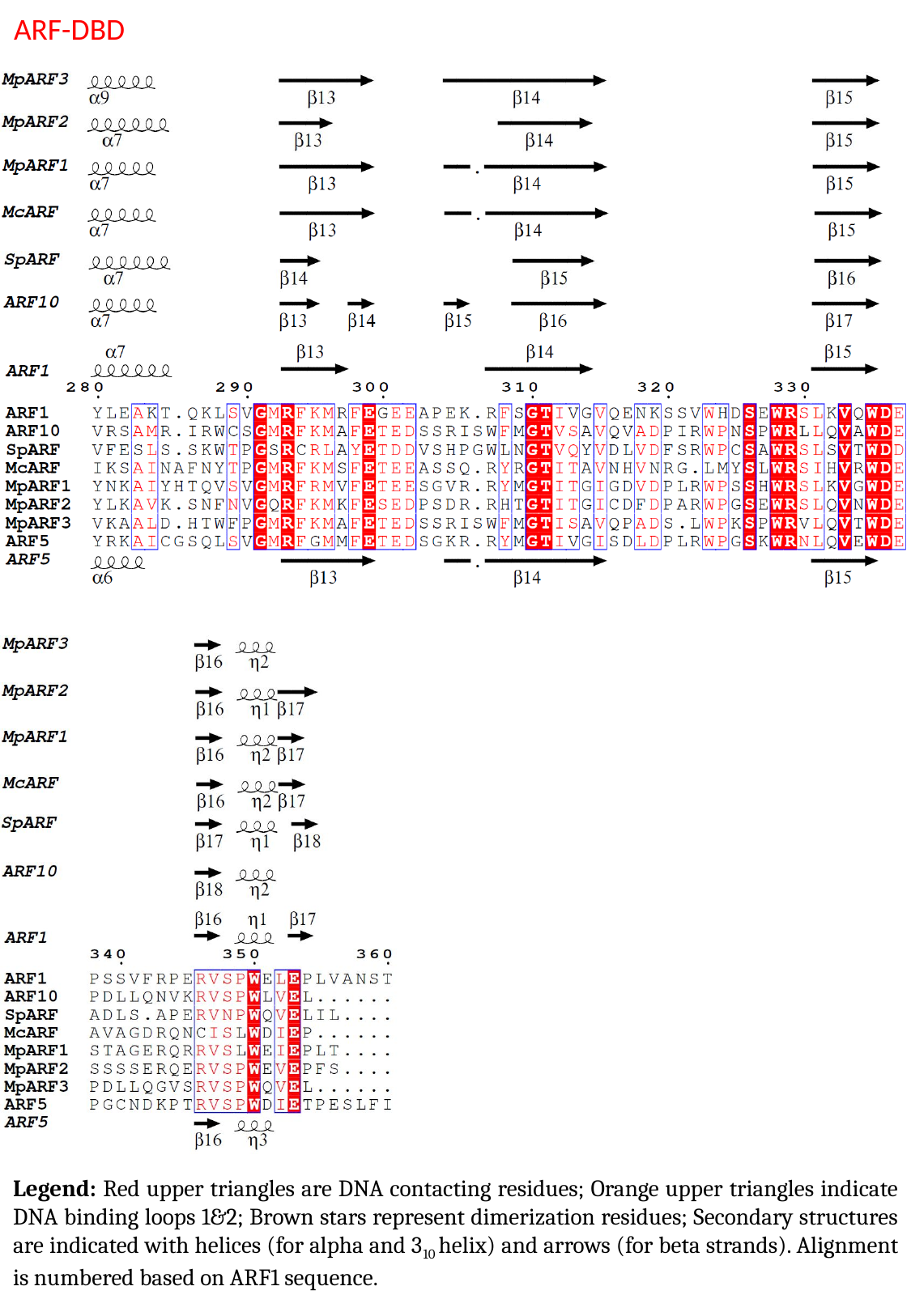

ARF-DBD
Legend: Red upper triangles are DNA contacting residues; Orange upper triangles indicate DNA binding loops 1&2; Brown stars represent dimerization residues; Secondary structures are indicated with helices (for alpha and 310 helix) and arrows (for beta strands). Alignment is numbered based on ARF1 sequence.

## Slide 7
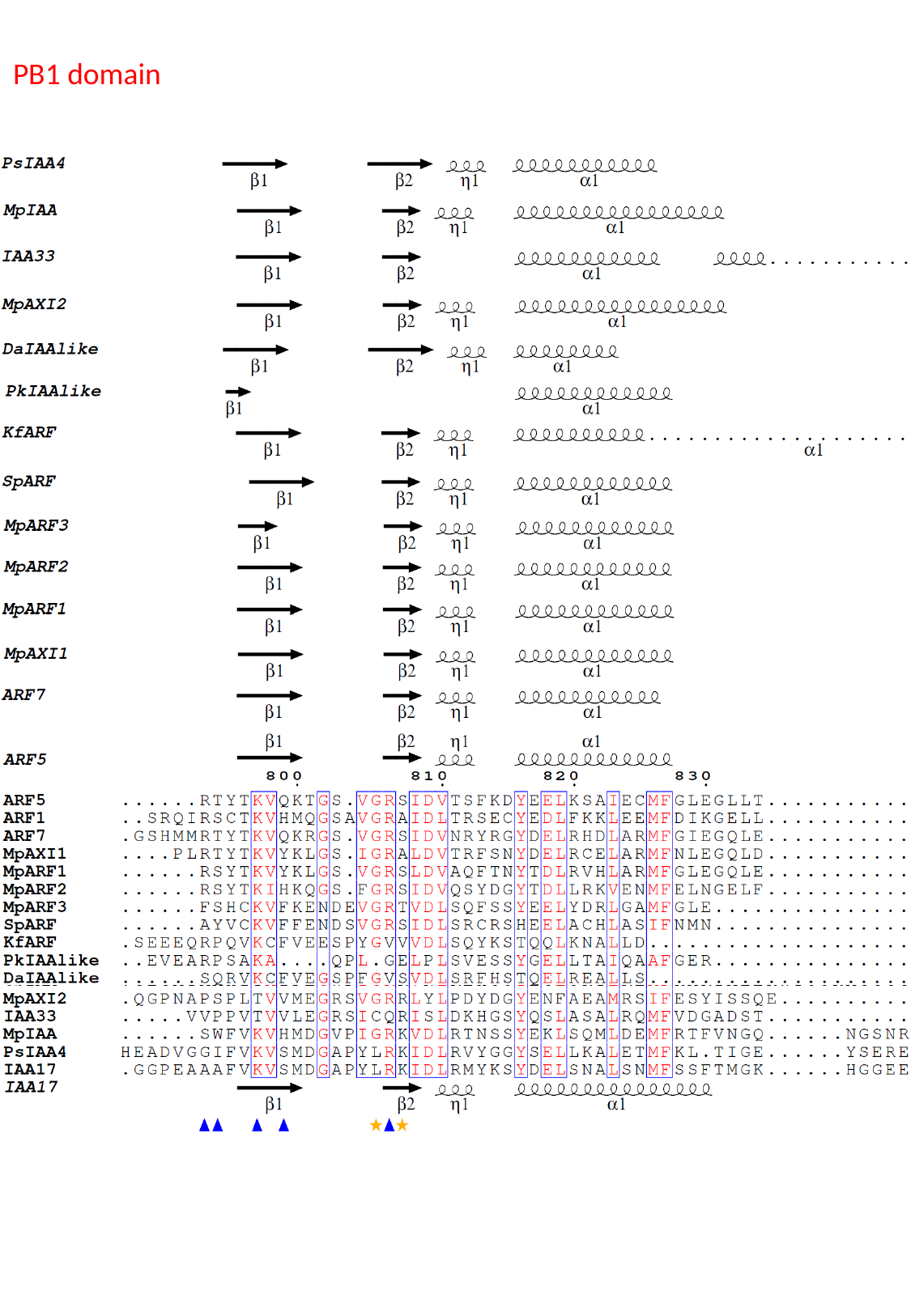

PB1 domain

## Slide 8
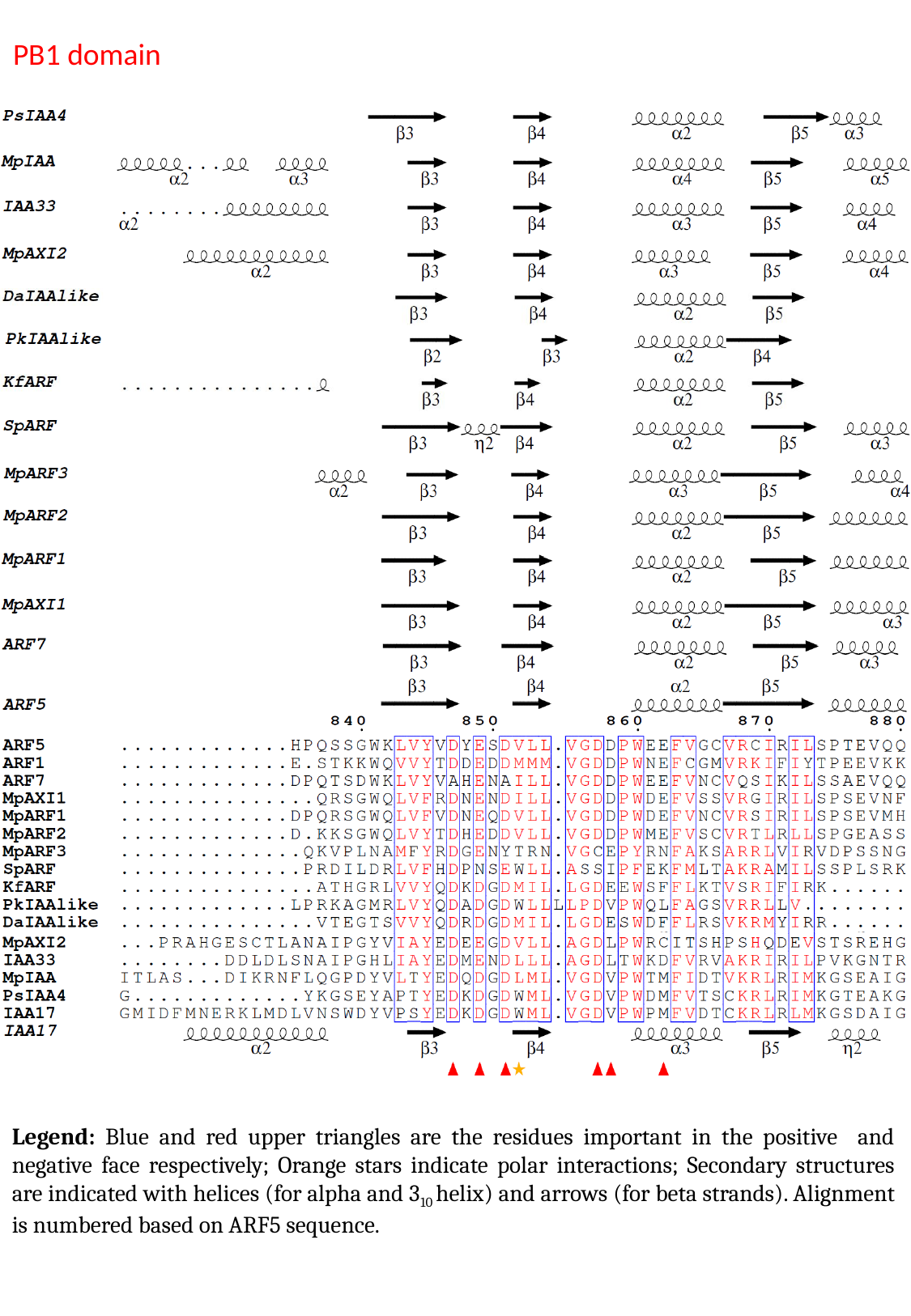

PB1 domain
Legend: Blue and red upper triangles are the residues important in the positive and negative face respectively; Orange stars indicate polar interactions; Secondary structures are indicated with helices (for alpha and 310 helix) and arrows (for beta strands). Alignment is numbered based on ARF5 sequence.

## Slide 9
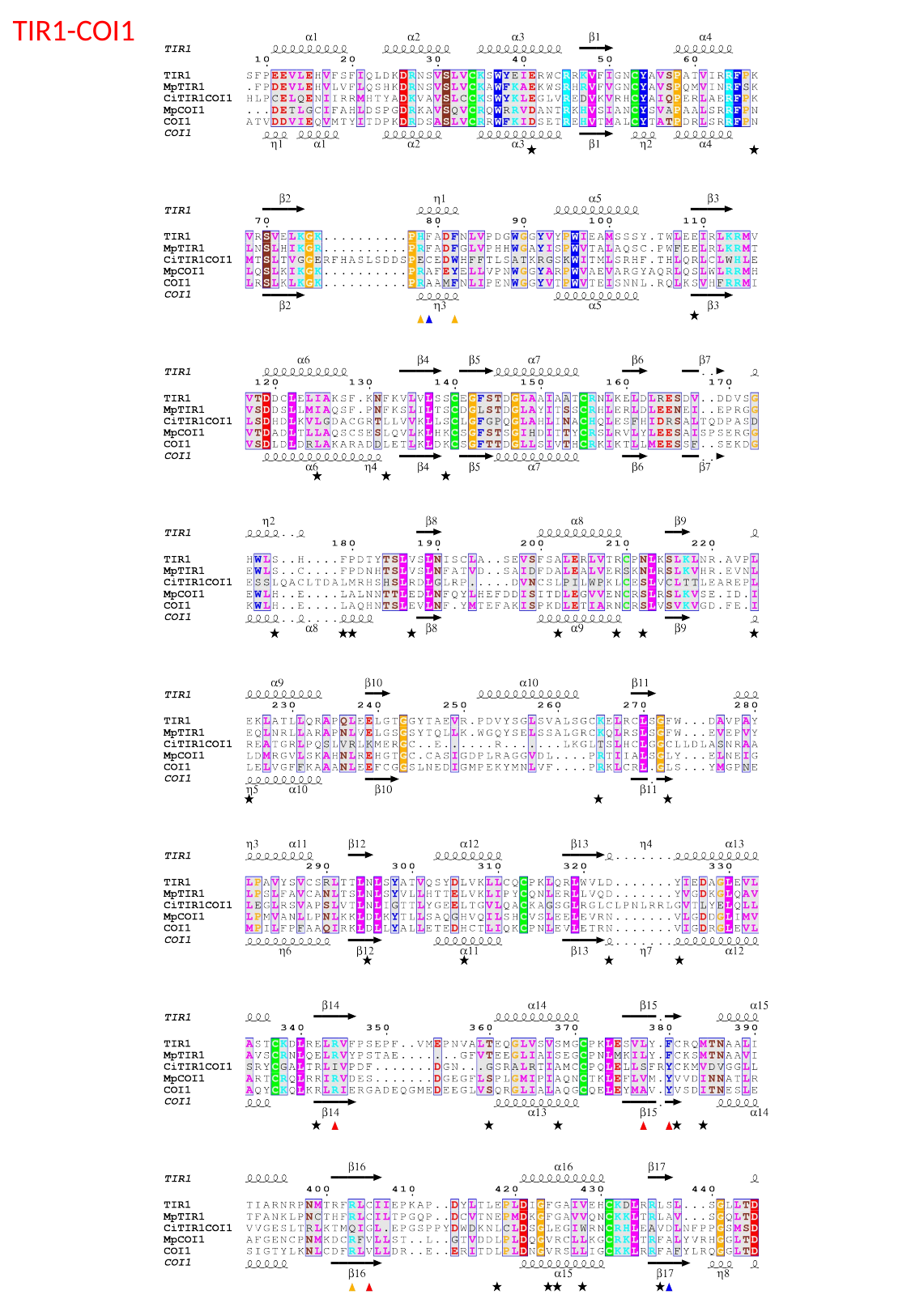

TIR1-COI1

## Slide 10
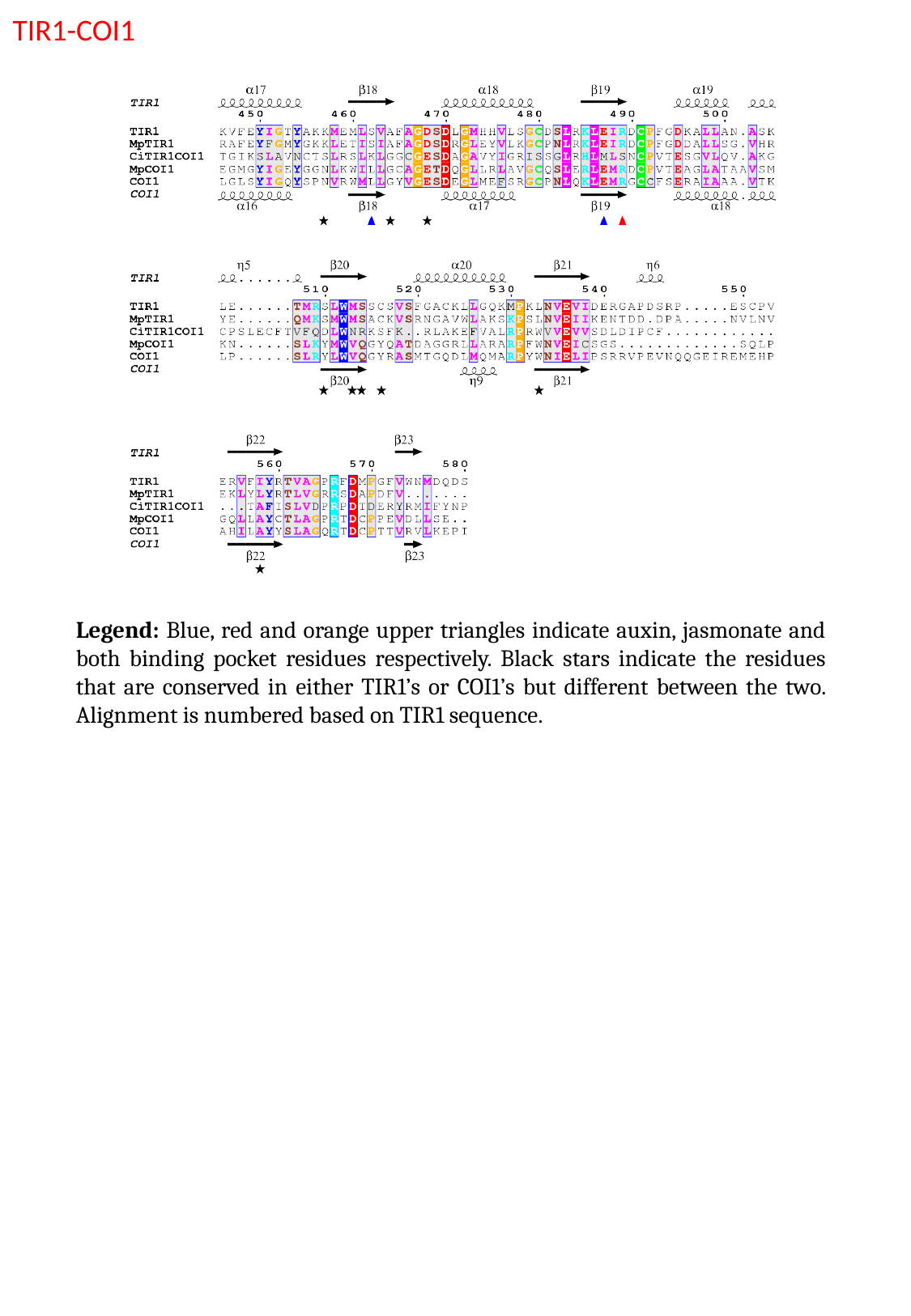

TIR1-COI1
Legend: Blue, red and orange upper triangles indicate auxin, jasmonate and both binding pocket residues respectively. Black stars indicate the residues that are conserved in either TIR1’s or COI1’s but different between the two. Alignment is numbered based on TIR1 sequence.
