# Supplementary material for: Origin and evolution of the nuclear auxin response system
Source: eLife. 2018 Mar 27;7:e33399. doi: 10.7554/eLife.33399 (PMC5873896; doi:10.7554/eLife.33399)
Supplement: Supplementary file 4. [file elife-33399-supp4.zip › web_session/index.html]

Cytoscape Session Viewer
