# Supplementary material for: Origin and evolution of the nuclear auxin response system
Source: eLife. 2018 Mar 27;7:e33399. doi: 10.7554/eLife.33399 (PMC5873896; doi:10.7554/eLife.33399)
Supplement: Supplementary file 4. [file elife-33399-supp4.zip › web_session/views/main.html]

### Network:

### Visual Style:

### Layout:

|  |  |  |  |
| --- | --- | --- | --- |
| SUID {{columnName}}|  |  | | --- | --- | | {{ node.id() }} {{node.data(colName)}} | | | |

|  |  |  |  |
| --- | --- | --- | --- |
| SUID {{columnName}}|  |  | | --- | --- | | {{ edge.id() }} {{edge.data(colName)}} | | | |
