# Supplementary material for: Origin and evolution of the nuclear auxin response system
Source: eLife. 2018 Mar 27;7:e33399. doi: 10.7554/eLife.33399 (PMC5873896; doi:10.7554/eLife.33399)
Supplement: Supplementary file 4. [file elife-33399-supp4.zip › web_session/404.html]

Page Not Found :( 

# Not found :(

Sorry, but the page you were trying to view does not exist.

It looks like this was the result of either:

- a mistyped address
- an out-of-date link
